# PARP inhibitor resistance: the underlying mechanisms and clinical implications

**DOI:** 10.1186/s12943-020-01227-0

**Published:** 2020-06-20

**Authors:** He Li, Zhao-Yi Liu, Nayiyuan Wu, Yong-Chang Chen, Quan Cheng, Jing Wang

**Affiliations:** 1 Hunan Clinical Research Center in Gynecologic Cancer, Hunan Cancer Hospital and The Affiliated Cancer Hospital of Xiangya School of Medicine, Central South University, 283, Tongzipo Road, Changsha, 410013 Hunan People’s Republic of China; 2 Department of Neurosurgery, Xiangya Hospital, Central South University, Changsha, 410013 Hunan People’s Republic of China; 3 Department of Gynecologic Cancer, Hunan Cancer Hospital and The Affiliated Cancer Hospital of Xiangya School of Medicine, Central South University, 283, Tongzipo Road, Changsha, 410013 Hunan People’s Republic of China

**Keywords:** PARPi, Homologous recombination, Resistance, Synthetic lethality

## Abstract

Due to the DNA repair defect, BRCA1/2 deficient tumor cells are more sensitive to PARP inhibitors (PARPi) through the mechanism of synthetic lethality. At present, several PAPRi targeting poly (ADP-ribose) polymerase (PARP) have been approved for ovarian cancer and breast cancer indications. However, PARPi resistance is ubiquitous in clinic. More than 40% BRCA1/2-deficient patients fail to respond to PARPi. In addition, lots of patients acquire PARPi resistance with prolonged oral administration of PARPi. Homologous recombination repair deficient (HRD), as an essential prerequisite of synthetic lethality, plays a vital role in killing tumor cells. Therefore, Homologous recombination repair restoration (HRR) becomes the predominant reason of PARPi resistance. Recently, it was reported that DNA replication fork protection also contributed to PARPi resistance in BRCA1/2-deficient cells and patients. Moreover, various factors, such as reversion mutations, epigenetic modification, restoration of ADP-ribosylation (PARylation) and pharmacological alteration lead to PARPi resistance as well. In this review, we reviewed the underlying mechanisms of PARP inhibitor resistance in detail and summarized the potential strategies to overcome PARPi resistance and increase PARPi sensitivity.

## Introduction

**Fig. 1:**
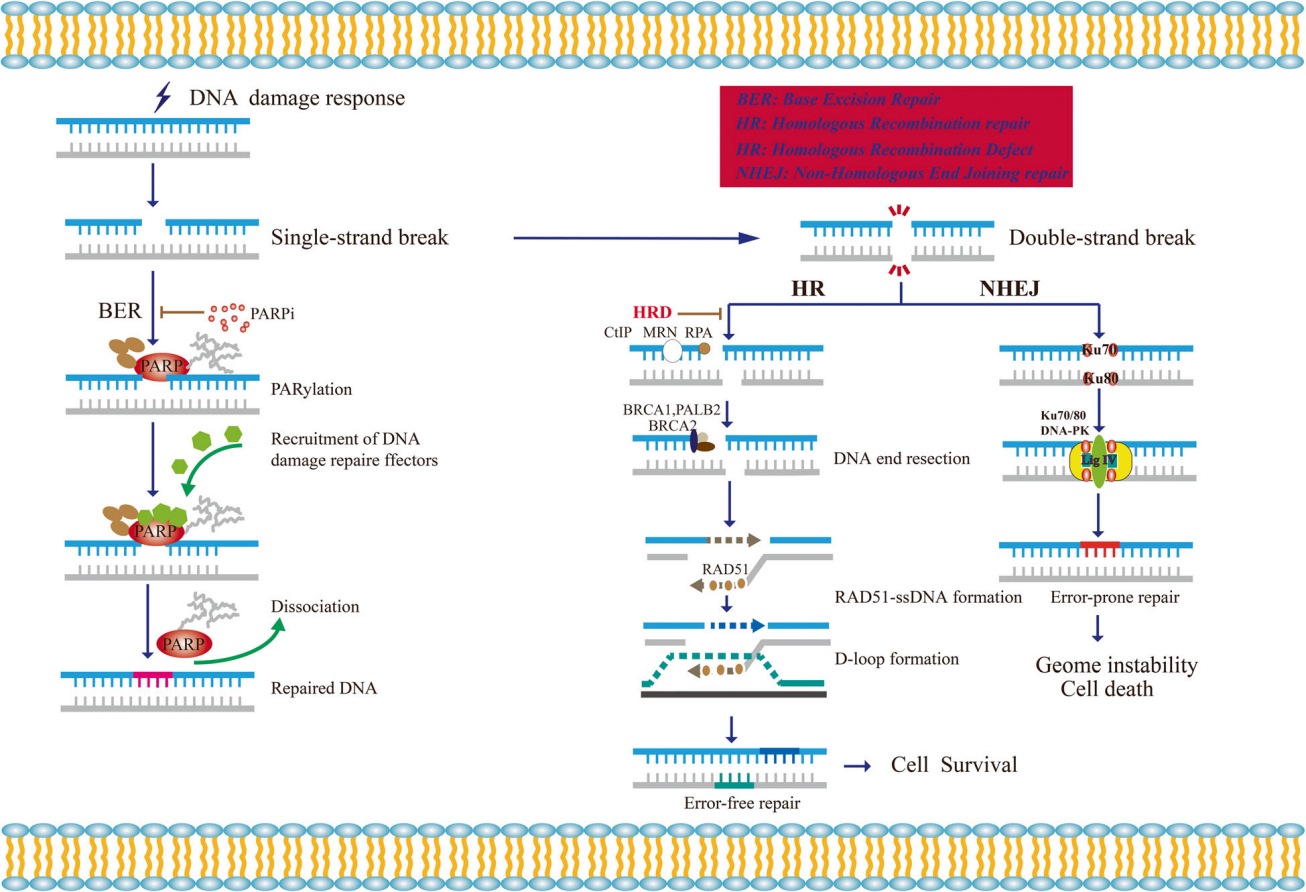
Schematic describing the function the principle of synthetic lethality interaction between PARPs and BRCA1/2. When cells suffer from DNA response, single-strand breaks emerge. PARPs, especially PARP1, bind to the DNA break sites, which result in the PARylation of target proteins and recrement of the DNA damage repair effectors. Then the auto-PARylation on PARPs leads to the dissociation of PARPs from DNA. Treating HR-deficient tumor cells with PARPi, NHEJ is the only pathway to use to repair double-strand break, which lead to accumulation of genome instability and cell death for the low fidelity

DNA damage response (DDR) is vital to maintaining genome stability [1]. When cells suffer from DNA damage, DDR is instigated and it can remove the damage by specified DNA repair pathways, including homologous recombination repair (HR), non-homologous end joining repair (NHEJ), single stranded break repair (SSBR) [2]. To cope with DNA single-strand breaks (SSB), base excision repair (BER) is activated in mammalian cells. Poly (ADP-ribose) polymerases (PARPs), especially PARP1, PARP2 and PARP3 are key to BER [3, 4]. As DNA damage sensors and signal transducers, they can bind damaged DNA at single strand DNA breaks sites, which result in the recruitment of DNA repair effectors to the sites of DNA breaks [4, 5]. NHEJ and HR are two mainly pathways to resolve the DNA double- strand breaks (DSB). NHEJ is an error prone pathway. In this mechanism, DSB sites are repaired by blunt end ligation with low fidelity [6]. While the use of NHEJ leads to accumulation of genetic aberrations, chromosomal instability, cell cycle arrest and apoptosis [7]. However, HR is a process of accurate restoration of the DSB with high fidelity [8]. BRCA1/2 proteins are crucial for the error-free repair of HR [9]. In the S/G2 phase, BRCA1 is recruited to the DSB sites, which counteracts 53BP1 and initiates ubiquitination of C-terminal binding protein interacting protein (CtIP) [10]. With the assistance of CtIP, the 5′ to 3′ resection occurs and generates 3′ overhangs. Afterwards, BRCA2 and PALB2 participate in the formation of the nucleoprotein filament and D-loop [11, 12] (Fig. 1). Given that DDR has the ability to overcome the cytotoxicity induced by chemo- and radiotherapy treatment, it’s important to uncover the underlying mechanisms of DNA repair pathway and exploit new drugs.

Germline mutations in BRCA1/2 (gBRCAm) predispose to ovarian cancer and breast cancer. Besides, somatic mutations of BRCA1/2 (sBRCAm) have also been suggested in various cancer types. Especially, nearly 20% of patients (16% gBRCAm and 4% sBRCAm) occur in ovarian cancer [13]. More importantly, up to 50% high-grade serious ovarian cancer (HGSOC) patients present as HRD [14]. Therefore, inhibition of PARPs may cause both SSBR deficient and HRD in BRCA1/2 deficient patients, leading to cell death [15, 16]. This is the so-called “synthetic lethality”, which is a concept proposed a century ago to describe the condition whereby a defect of either one of two genes have no/little effect but the combination of both genes (BRCA and PARPs) lead to cell death [17].

PARPi are the first agents designed to exploit synthetic lethality and permitted to use in clinic. They have the ability to bind and trap PARPs on DNA, preventing the release of PARPs from DNA break sites and removing PARPs from their normal catalytic cycle [5]. Due to more benefits and less adverse effects, olaparib (lynparza), niraparib (ZEJULA) and rucaparib (RUBRACA) are indicated for the maintenance treatment of recurrent ovarian cancer patients, who are in a complete or partial response to platinum-based chemotherapy in United states [18–21]. Olaparib is also approved to treating gBRCAm advanced ovarian cancer as four lines of chemotherapy [18]. It can also be used to treat gBRCAm, HER2-negative metastatic breast cancer patients, who have been treated with chemotherapy in the neoadjuvant, adjuvant, or metastatic setting [22, 23]. Recently, it’s suggested that carriers with HRD but not gBRCAm or sBRCAm, which is termed as “BRCAness”, are also sensitive to PARPi [24]. However, BRCA1/2 mutations remain the strongest genetic predictor of sensitivity of PARPi [25].

Similarity with other chemotherapy agents, PARPi also faced the drug resistance. More than 40% of BRCAm ovarian cancer patients failed to benefit from PARPi [26, 27]. Considering the important roles of HR repair pathway and protection of stalled replication forks in the effect of PARPi, we described the effects of DNA repair response and protection of stalled replication forks on PARPi resistance in detail. Besides, we reviewed the association between PARPi resistance and other factors, such as reversion mutations, epigenetic modification, restoration of PARPylation and pharmacological alteration. Finally, we summarized the feasible strategies to overcome PARPi resistance and enhance PARPi sensitivity in clinic.

### Restoration of HR repair in PARPi resistance

**Fig. 2:**
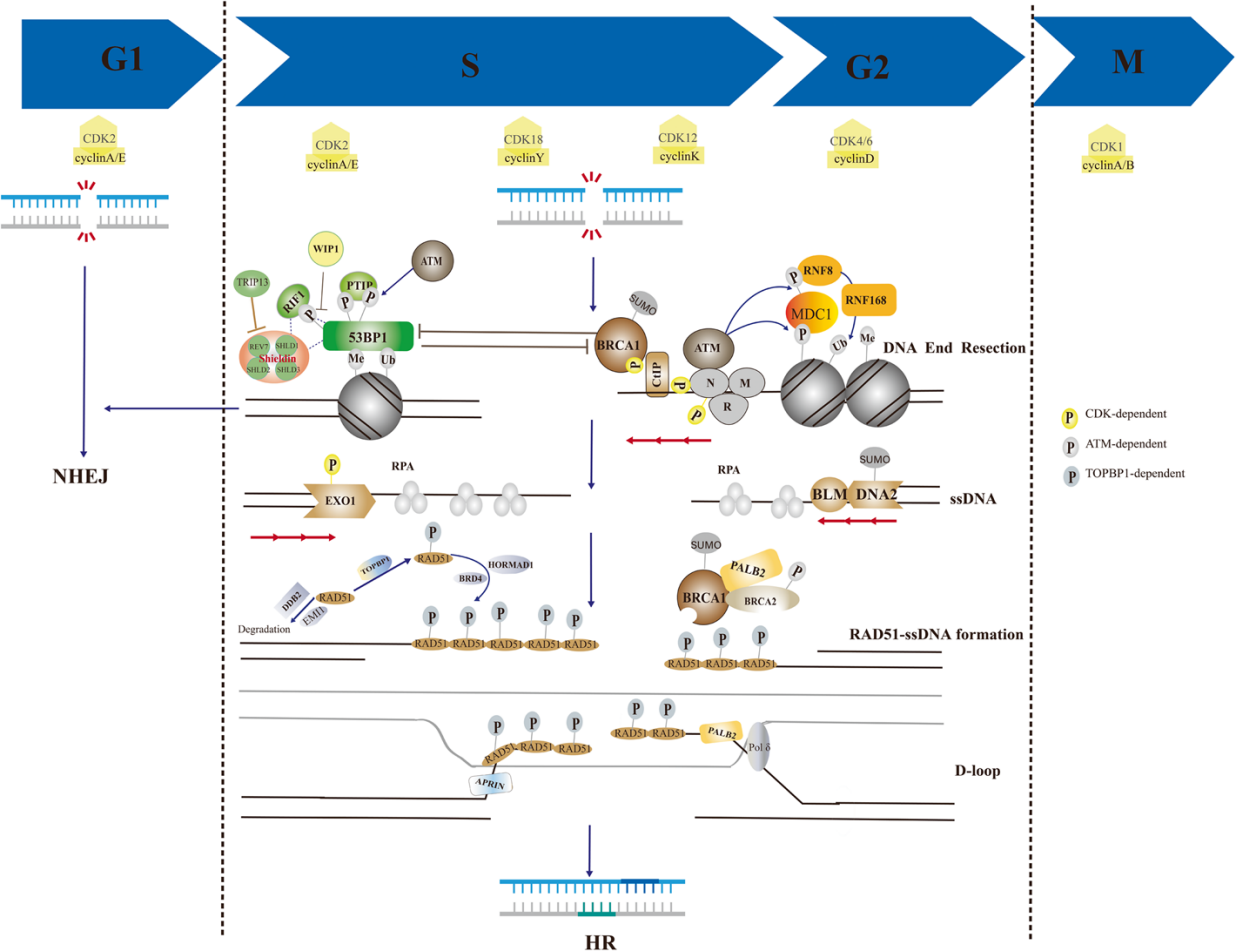
Homologous recombination repair in S/G2 phase. The double-strand break ends are resected by MRE11-RAD50-NBS1(MRN) complex together with CtIP. ATM is recruited to DSBs through MRN and phosphorylates targets such as 53BP1 and MDC1. MDC1 phosphorylation recruits the E3 ubiquitin ligase RNF8, which, through recruitment of a second E3 ubiquitin ligase (RNF168), leads to histone H2A ubiquitylation. This modification, together with H4K20 methylation, allows for 53BP1 recruitment. 53BP1 phosphorylation allows its interaction with RIF1 and PTIP, which can be blocked by WIP1. 53BP1 blocks DNA resection by recruiting shieldin and presents cells to NHEJ. While, BRCA1 counteracts the protection function of 53BP1, leading to the resection of DNA ends. Afterwards, the resected DNA ends are coated by PRA. With the favor of PALB2, BRCA2 binds with BRCA1 and promotes the loading of RAD51. The RAD51 mediates the invasion of the homologous sequence and formation of the nucleoprotein filament and D-loop by eliminating secondary structure formation. EMI and DDB2 mediate the degradation of RAD51. TOPBP1 phosphorylates RAD51. BRD4 and HORMAD1 are key regulators of RAD51 accumulation on chromatin. P, phosphorylation; Ub, ubiquitylation; Me, methylation, SUMO, SUMOylation, red arrows, resection

Due to HRD is the main premise of anticancer effects of PARPi, it is crucial to understand the HR repair pathway. When DSB happen in mammalian cells, the DDR is activated. Coordinately, cells employ two typical mechanisms to repair DSB: HR and NHEJ. Normally, NHEJ is the mainly repair mechanism by ligating the broken DNA ends in a nonhomologous end-joining way and occurs throughout the cell cycle, especially in G0/G1 phase. However, HR predominates the S/G2 phase, due to the high DNA replication and available sister template [28]. In the process of HR, the DSB ends are firstly resected by Mre11-Rad50-Nbs1(MRN) complex together with CtIP and nucleases (EXO1, DNA2 and MUS8), leading to the formation of the single-strand DNA (ssDNA) and committing the cells to HR [29]. Afterwards, the resected DNA ends are coated by hyperphosphorylated single-strand DNA binding protein A (RPA) [30]. The variant H2AX (named γH2AX) is activated and phosphorylated by apical kinases, such as ataxia-telangiectasia mutated (ATM) and ATM- and Rad3-related (ATR). The spreading of γH2AX along the chromosome assists the recruitment and accumulation of additional DDR proteins, including p53-binding protein (53BP1) and BRCA1 to the DDR foci [31]. With the favor of PALB2, BRCA2 binds with BRCA1 and promotes the loading of recombinase RAD51 on the ssDNA [11]. The RAD51 mediates the invasion of the homologous sequence and formation of the nucleoprotein filament and D-loop by eliminating secondary structure formation and protecting DNA ends from degradation [32] (Fig. 2). Therefore, the restoration of HR pathway by inducing the process of DNA end resection and formation of nucleoprotein filament and D-loop may lead to PARPi resistance.

### DNA end resection in PARPi resistance

Considering that DNA end resection is the key of different DNA repair pathways choices, it’s likely that DNA end resection dictates the different repair outcome and PARPi sensitivity. Recently, multiple reports have suggested that DNA end resection participated in the PARPi resistance (Fig. 2).

Cell cycle controls the choice of DSB repair pathways [33]. In the G1 phase, 53BP1 and RIF1 proteins localize to DSB sites, leading to the inhibition of BRCA1 recruitment, blocking DNA resection and promoting NHEJ repair pathway. Otherwise, DNA end resection is stimulated in the phase of S/G2 phase and promotes HR repair [34]. It is worth mentioning that DNA end resection is depended on cyclin-dependent kinases (CDKs) activity, which mediate phosphorylation of MRN complex and CtIP [35, 36]. It was reported that CDK5-silenced Hela cells were more sensitive to PARPi [36]. Besides, CDK12 was identified as a determinant of olaparib in the models of HGSOC by genome-wide synthetic lethal screen [37]. Loss-of functions (LOF) mutations in CDK12 disrupted HR repair and sensitized ovarian cancer cells to veliparib [38]. In triple-negative breast cancer (TNBC), deletion of CDK12 reversed both primary PARPi resistance and secondary PARPi resistance, no matter in BRCA wild-type and mutated models [39]. Besides, CDK18 facilitates ATR activation by interacting with ATR and regulating ATR-Rad9/ATR-ETAA1 interactions, promoting HR and PARPi resistance in glioblastoma stem-like cells [40]. Recently, a case report results indicated that PARPi combined with CDK4/6 inhibitor (palbociclib) revealed more excellent therapeutic effects than PARPi alone in the treating with BRCA-mutated, ER-positive breast cancer [41]. All these evidences suggested that CDKs blocked DNA end resection and lead to PARPi resistance and its inhibitors might overcome the PARPi resistance. Prospectively, the combination therapy of PARPi and CDKs inhibitors is applied in clinic.

In addition to Cell cycle and CDKs, accessory factors including 53BP1, REV7 and RIF1, contribute a lot to DNA end resection and PARPi resistance [42–44]. 53BP1, which is a chromatin-binding protein, blocks DNA resection by preventing the accession of CtIP to the DSB sites [45]. It has been suggested that the loss of 53BP1 induced DNA end resection and HR restoration, leading to PARPi resistance in various cancers, such as breast cancer [42], glioblastoma [46] and ovarian cancer [47]. Mainly, 53BP1 protects DNA ends from resection in two ways. One way is to strengthen the nucleosomal barrier to end-resection nucleases by recognizing and binding to the nucleosomes containing H4K20m2 and H2AK15ub [48]. The other way is to recruit effector complex proteins with end-protection activity^49^. Recently, it was demonstrated that shieldin, an effector complex composed by SHLD1, SHLD2, SHLD3 and REV7, were recruited by 53BP1 to the DSB sites in a 53BP1 and RIF1 depend manner [49]. Numerous evidences revealed that shieldin, as the key regulator of NHEJ repair and HR repair, was also associated with PARPi resistance [49–51]. REV7, as the component of shieldin, was also suggested to counteract DNA end resection and sensitize cells to PARPi [43]. Likewise, catalysed the inactivating conformational change of REV7 and dissociated REV7-Shieldin by TRIP13 ATPase promoted HR, leading to PARPi resistance [52]. The protection function of 53BP1 requires the interactions of PTIP and RIF1, which is depends on ATM [44, 53]. Hence, the interaction between 53BP1 and RIF1 plays pivotal roles in DNA end resection and PARPi resistance. As is known to us, only when 53BP1 is phosphorylated by ATM can it recruit RIF1 and PTIP [54]. It was demonstrated that ATM-deficient cancer cells was more sensitive to PARPi than ATM-proficient cells and the combination use of ATM inhibitors enhanced PARPi efficacy [55, 56]. Besides, multiple clinical trials results indicated that patients with low ATM proteins had a greater benefit from PARPi and more favorable prognosis [57–59]. Recently, it was disclosed that WIP1 dephosphorylated 53BP1 at Threonine 543 and attenuated its interaction with RIF1, leading to decreased sensitivity of cancer cells to PARPi [60], which confirmed the importance of the interaction between 5BP1 and RIF1 once more. Obviously, nucleases (i.e., MRE11 [61–63], DNA2 [64] and EXO1 [65, 66]), functioning as “DNA end clipping” in the process of DNA end resection, affected the sensitivity and resistance of PARPi.

### Formation of RAD51-ssDNA filament and D-loop in PARPi resistance

The RAD51-ssDNA filament performs the central functions in homology search, DNA stand exchange and HR repair (Fig. 2). Especially, RAD51 foci is suggested to serve as a functional biomarker of HR repair and PAPRi resistance beyond BRCA mutation [67–69]. In the issue, the balance between RAD51 filament formation and disruption seem particularly important. By using a genetic screen, EMI1 was identified to constitutively target RAD51 for degradation and function as a modulator of PARPi sensitivity. Downregulation of EMI1 enhanced the RAD51 accumulation, leading to restoring HR and developing PARPi resistance in BRCA1-deficient TNBC cells [70]. Similarly, DNA damage binding protein 2 (DDB2), a DNA damage recognition factor, was reported to participate in the regulation of RAD51 degradation by physical interaction in TNBC cells. The inhibition of DDB2 induced RAD51 polyubiquitination and proteasomal degradation, leading to defective HR and sensitivity to PARPi [71]. Topoisomerase IIβ-binding protein 1 (TOPBP1) was essential for RAD51 phosphorylation at serine 14, which was necessary for RAD51 recruitment on chromatin and formation of RAD51 foci. Absent of TOPBP1 abrogated the HR and increased sensitivity of ovarian cancer cells to olaparib [72]. Bromodomain protein 4 (BRD4) is a kind of key chromosomal regulator of genome stability. The inhibition of BRD4 recruited RAD51 accumulation without activation of ATM/ATR-dependent DNA damage response [73]. It was mentioned that BRD4 was amplified in various cancer [74]. Growing evidence suggested that BRD4 inhibitors (JQ1, INCB054329) sensitized to PARPi and expanded the utility of PARPi in clinic [74–77]. In human lung adenocarcinoma (LUAD) tumors, patients expressing high HORMAD1 exhibited elevated mutational burden and poor survival. HORMAD1 were enriched for genes essential HR and promoted RAD51 filament formation. Accordingly, high expression of HORMAD1 contributed to PARPi resistance [78]. APRIN and PALB2 preferentially bind to D-loop structures and directly interact with RAD51 to stimulate strand invasion and promote HR. It has been shown that deletion of APRIN and PALB2 induced “BRCAness” and sensitized cells to PARPi [79, 80]. Moreover, Pol δ played vital roles in D-loop extension and inhibition of Pol δ also enhanced the sensitivity of HR-proficient cancer cells to PARPi [81].

### Reversion mutations in PARPi resistance

In 2008, the influence of reversion mutations on PARPi resistance was independently discovered by two groups. Ashworth et al derived PARPi-resistant clones by deleting the BRCA2 c.6174delT frameshift mutation of human CAPAN1 pancreatic cancer cell line, a BRCA2-deficient cell line. Consequently, the reconstituted BRCA2-deficient cells acquired PARPi resistance [82]. Meanwhile, Sakai et al demonstrated that secondary mutations restored the wild-type BRCA2 reading frame was a major clinical mediator of acquired resistance to platinum and PARPi [83]. By using liquid biopsy or circulating cell-free DNA (cfDNA), lots of BRCA reversion mutations have been discovered to restore the open reading frame (ORF) of BRCA1/2 and confer resistance to PARPi-based therapy in various cancers [84–90] (Table 1).

**Table 1:** Reversion mutations (variant allele fraction > 0.5%) conferred resistance to PARPi resistance

Gene	Primary mutations	Reversion mutations	Variant allele fraction		Cancer type
			plasma	tumor	
BRCA1	Q1756fs*74 (c.5266dupC)	Q1756_D1757 > PG (c.5263_5272 > TCCCCAGGAC)		3.2%	HGPSC^a^
BRCA1	1479delAG (c.1360_1361del)	s454_l467del (c.1361_1402del)			TNBC^b^
BRCA2	K2162fs*5 (c.6486_6489delACAA)	K2150fs^a^17 (c.6448_6473del26)		8%	mPC^c^
BRCA2	V1283fs*2 (c.3847_3848delGT)	D1280_N1288del (c.3838_3864del27)	33%	57%	Breast cancer
BRCA2	V1804Kfs (c.5410_5411del)	Y1480_A1896del (c.4434_5686delinsTT)	0.60%		mPC^c^
BRCA2	V1804Kfs (c.5410_5411del)	I1633_I2269del (c.4897_6807del)	0.40%	2.80%	mPC^c^
BRCA2	Q2960X (c.9106C > T)	Q2960E (c.9106C > G)		67%	Breast cancer
BRCA2	E1493Vfs*9 (c.4705_4708delGAAA)	I1490_E1493del (c.4698-4709delAAATACTGAAAG)		55–56%	HGPSC^a^
BRCA2	S1982fs (c.5946delT)	S1982_ A1996del (c.5946_5990del45)		1%	Prostate cancer
BRCA2	S1982fs (c.5946delT)	S1985fs (c.5949_5952dupAAAA)		0.5%	Prostate cancer
BRCA2	N1910fs*2 (5727_5728insG)	A1843_S1985del (5528_5956del429)		0.53%	prostate cancer
BRCA2	N1910fs*2 (5727_5728insG)	A1891_M1936del (5671_5808del138)		0.54%	prostate cancer
BRCA2	N1910fs*2 (5727_5728insG)	D1909_D1911 > EDY (5727_5731TAATG > AGACT)		0.63%	prostate cancer
BRCA2	N1910fs*2 (5727_5728insG)	L1908_S1917del (5721_5750del30)		1.8%	prostate cancer
BRCA2	N1910fs*2 (5727_5728insG)	N1766_Q2009del (5292_6025 > CA)		1.3%	prostate cancer
BRCA2	N1910fs*2 (5727_5728insG)	N1910_D1911del (5728_5733delAATGAT)		3.3%	prostate cancer
BRCA2	N1910fs*2 (5727_5728insG)	S1788_P2114 > DTT (5362_6340 > GATACCA)		1.2%	prostate cancer
BRCA2	N1910fs*2 (5727_5728insG)	NA (splice site 5333_6841 + 197del1706)		4.8%	prostate cancer

*HGPSC: High-grade papillary serous carcinoma;

*TNBC: triple-negative breast cancer

* mPC: metastatic pancreatic cancer;

NA: unknown

Full length BRCA1 consists of N-terminal domains (BRCT), N-terminal RING domain and coiled-coil domain. BRCT is responsible for binding phosphorylated proteins such as CtIP. N-terminal RING domain can stabilize BRCA1 and ensure the E3 ligase activity [91]. Multiple evidence suggested that reversion mutations, which restored the functions of BRCT and N–terminal RING domain, played essentials roles in PAPRi resistance [92–94]. In addition, cancer cells lacking the exon 11 of BRCA1 promoted partial PARPi resistance [95]. BRCA2 contains a DNA-binding domain and eight BRC repeats that bind to RAD51 and mediates the recruitments of RAD51 and strand exchange in HR [91]. It was suggested that each BRC repeats was divided into two categories and only BRC 1–4 bound to RAD51 with high affinity and enhanced DNA strand exchange while BRC 5–8 bound to RAD51 with low affinity and did not affect DNA strand exchange [96]. However, an in vitro study indicated that BRCA2 mutations lacking BRC 6–8 also lead to PARPi resistance [82]. Recently, two reversion mutations (c.4434_5686delinsTT and c.4897_6807del) produced truncated BRCA2 protein were thought to be competent in conferring PARPi resistance [89]. In addition to reversion mutations in BRCA1/2, Secondary somatic mutations restoring Rad51C and Rad51D were also demonstrated to be associated with acquired resistance to the PARPi [84]. With the development of gene editing, CRISPR-Cas9 screens were recently used to identify point mutations in PARP1 conferring PARPi resistance. Several mutations in PARP1 including p.R591C and p.848delY, were identified to cause PARPi resistance. More importantly, the CRISPR-Cas9 “tad-mutate-enrich” mutagenesis screens approach could be employed in the analysis of other gene mutations [97].

Taken together with the growing body of data identifying reversion mutations in PARPi resistance, it seems to be the most well-validated mechanism of PARPi resistance in BRCAm cancer patients. However, we must notice that whether the reversion mutations are induced by PARPi itself or other anticancer drugs or even spontaneous is unclear. After all, cancer cells harboring BRCA mutations prefer to NHEJ repair, which lead to accumulation of genetic aberrations and increased risk of reversion mutations. Moreover, before or even during treating with PARPi-based therapy, other anticancer drugs, such as platinum, were also administered to patients, which invisibly make the study more difficult to investigate the influence of PARPi-based therapy on secondary mutation in clinic.

Furthermore, the frequency of reversion mutations occurred among patient population is still known. Recently, the prevalence of BRCA reversion mutations in metastatic castration-resistant prostate cancer (mCRPC) was estimated. By using a large genomic database, 24 gBRCAm carriers were selected from 1534 patients with mCRPC underwent ctDNA testing. At the time of the blood draw, 5 of these 24 patients were given either a PARP inhibitor or platinum-based chemotherapy. Two patients, one receiving olaparib and one carboplatin, had BRCA2 reversion mutations. Therefore, in this germline mutation–positive, platinum- or PARP-exposed cohort, the frequency of BRCA2 reversion mutations was 40% [98]. However, another clinical trial result showed that 8 of 97 HGSOC patients with gBRCAm or sBRCAm (8.2%) were identified to have BRCA reversion mutations before treating with rucaparib. After treating with rucaparib, only 8 of 78 postprogression patients had BRCA reversion mutations and the occurrence rate of reversion mutations was only 10.3% [99]. All these results reflected that the BRCA reversion mutations might be different in various cancers. Due to the small sample size, additional studies with more patients and various cancers are needed to carry out.

### Protection of DNA replication fork in PARPi resistance

**Fig. 3:**
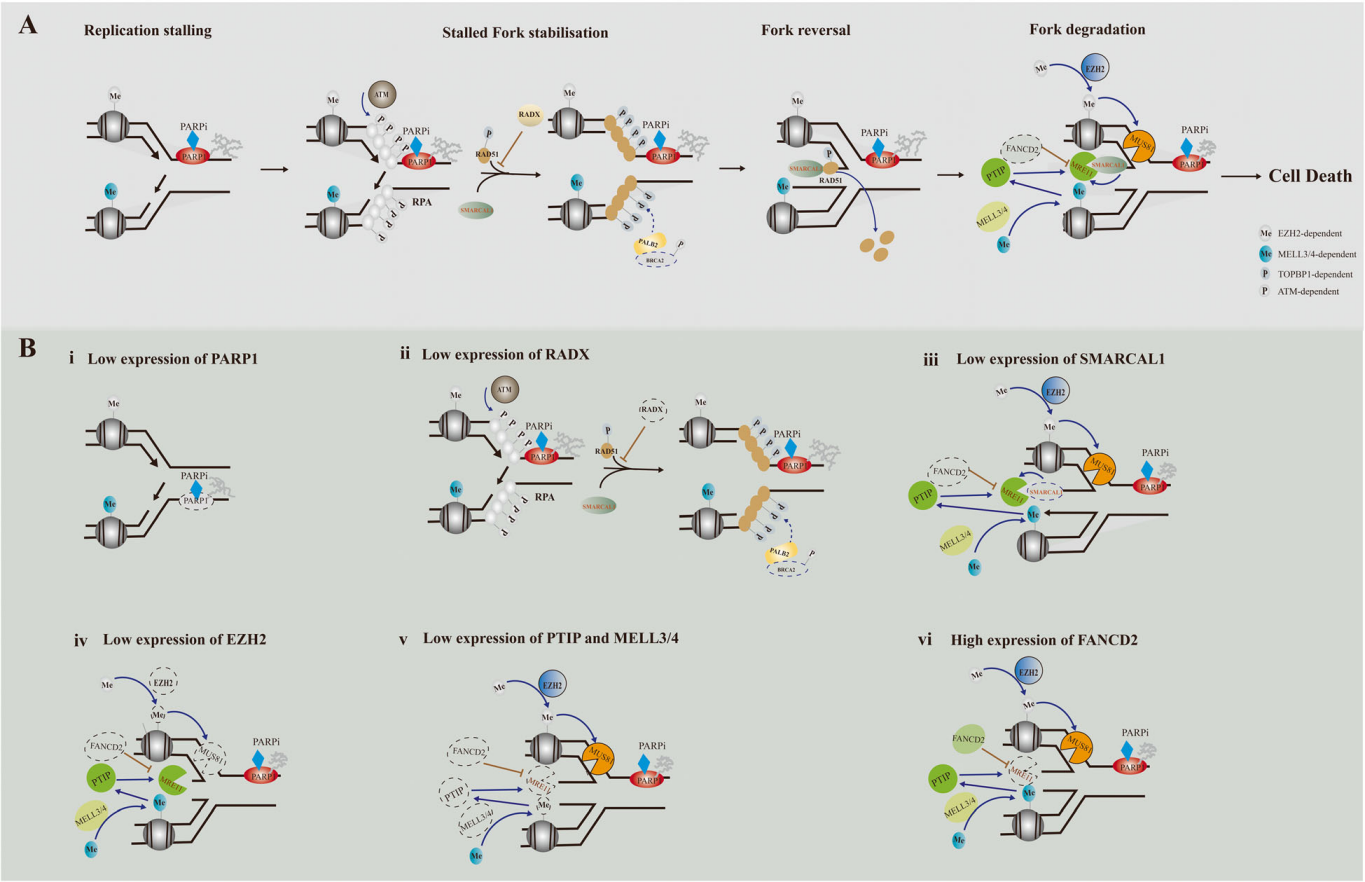
Schematic describing the function of PARP1 and BRCA1/2 in protection of DNA replication fork and mechanisms of protection of DNA replication fork leading to PARPi resistance. **a** PARPi trap PARP1 on DNA and cause fork stalling. After fork stalling, PRA is phosphorylated and ssDNA is coated by PRA rapidly. Then, RAD51 replaces RPA and mediates replication fork reversal. The revered fork can be degraded by MRE11 and MUS81. BRCA1/2 is relied on to protect nascent DNA replication forks from degradation. EZH2 induces H3K27 methylation and MUS81 recruitment at stalled forks. MELL3/4 induces H3K4 methylation increases the accumulation of PTIP, which leads to the recruitment of MRE11. FANCD2 suppresses MRE11-mediated fork degradation, which can be reversed by SMARCAL1. RADX blocks the recruitment of RAD51 at replication forks. **b** In BRCA1/2-deficient cells, low expression of PARP1, RADX, SMARCAL1, EZH2, PTIP, MELL3/4 and high expression of FANCD2 confer resistance of PARPi

In addition to DNA repair. PARP1 and BRCA1/2 participate in DNA replication. PARP1 has a key role in mediating the accumulation of regressed forks and avoiding an untimely restart of reversed forks, leading to DSB formation [100]. Both BRCA1 and BRCA2 protect nascent DNA at stalled replication forks from MRE11/DNA2-dependent degradation [101, 102]. When PARP inhibitors trap PARP on DNA to block DNA replication, cells will rely on BRCA1/2 to stabilize their stalled replication forks and prevent them from being extensively degraded by nucleases (i.e., MRE11, DNA2, MUS81). As BRCA1/2 is defective, the absence of DNA replication forks protection leads to genome instability and cell death [103] (Fig. 3). Recently, more and more evidence suggested that DNA replication fork protection but not HRR caused PARPi resistance in BRCAm cells and patients, which challenged the HR dominance in synthetic lethality (Fig. 3). Rondinelli et al. showed that low EZH2 levels reduced H3K27 methylation, prevented MUS81 recruitment at stalled forks and caused fork stabilization, which promoted PARPi resistance in BRCA2-deficient cells but not in BRCA1-deficient cells [104]. Besides, Ray et al. demonstrated that PTIP, MELL3/4 and CHD4 deficiency did not restore HR activity at DSB. Instead, their absence inhibited the recruitment of the MRE11 nuclease to stalled replication forks and protected nascent DNA strands from extensive degradation, which in turn lead to acquisition of PARPi resistance in BRCA2-deficient cells [105]. FANCD2 suppresses MRE11-mediated fork degradation in a manner dependent on nucleoprotein filaments and plays an important role in the stabilization of stalled replication forks [106]. It’s reported that FANCD2 was highly expressed in BRCA1/2-mutated breast cancer, ovarian cancers and uterine cancers. FANCD2 overexpression conferred resistance to PARPi in BRCA1/2-mutated breast cancer cell lines [107, 108]. Due to the DNA translocase activity, SMARCAL1, a member of SNF2 family, could reverse the nascent DNA degradation induced by FANCD2 deficiency in BRCA1/2-mutated breast cancer cells. It promoted the formation of ssDNA gaps at replication forks and reversed forks catalyzed by SMARCAL1 was prone to be degraded by MRE11. More importantly, its deletion promoted PARPi and cisplatin resistance [109]. In addition to SMARCAL1, the SNF2-famlily DNA translocases ZRANB3 and HLTF exhibited fork-remodeling activities similar to SMARCAL1, indicating that they might be associated with PARPi resistance as well [110]. RADX deletion restored fork protection but not HR by regulating RAD51 at replication forks and conferred PARPi resistance in BRCA2-mutated cancer cell lines [111]. These collective results refocus our PARPi resistance spotlight onto fork protection, which might make significant contributions to PARPi resistance [112]. Consequently, it might provide us a novel strategy in considering the future cancer therapy.

Stalled replication forks are a major source of genome instability in proliferating cells, which need to be stabilized or restarted to promote cell survival. Through decades’ efforts, multitude of mechanisms were found to protect stalled replication forks to preserve genome stability under replication stress. Except for the pathways mentioned above, RecQ helicases and pathways involved in ATR/CHK1-dependent checkpoint activation also play essential roles in replication fork protection and genome stability maintenance [103]. Therefore, they might function as part of mechanisms of PARPi resistance. However, there is no relevant preclinical and clinical studies up to now, which are expected to be taken into consideration in the future.

### Epigenetic modification, restoration of PARylation and pharmacological alteration in PARPi resistance

Epigenetic modification may affect PARPi sensitivity and lead to PARPi resistance. Multiple lines of treatment prior PARPi lead to loss of BRCA1 promoter methylation, which rescued the expression of BRCA1 and conferred resistance of PARPi [113]. MiR-622 and miR-493-5p induced PARPi resistance by suppressing NHEJ reapir and impacting multiple pathways pertinent to genome stability, respectively [114, 115]. Deubiquitination of BARD1 BRCT domain by USP15 assisted BRCA1 retention at DSBs and causes PARPi resistance [116]. Moreover, similar to deletion of 53BP1, acetylation of 53 bp1 inhibited NHEJ and promoted HR by negatively regulating 53 bp1 recruitment to DSBs, which made BRCA1-deficient cells acquire resistance to PARPi [117]. The role of N^6^-methyladenosine (m^6^A) modification in PARPi resistance was recently explored. Even though that there was no difference in total m^6^A-modified mRNA between parental and PARPi-resistant ovarian cancer PEO1 cells, the increased expression and N^6^-methylation modification of FZD10 were confirmed in resistant PEO1 cells. FZD10 contributed to PARPi resistance by upregulating the Wnt/β-catenin pathway [118].

As is known to us, PARPi kill tumor cells via PARPs activity inhibition and PARP trapping. PARPs activity increase and restoration of PARPylation are responsible to PARPi resistance. Phosphorylation of PARP1 at Tyr907, mediated by c-Met, increased PARP1 enzymatic activity and reduced its binding to PARPi, thereby rendering cancer cells resistant to PARPi [119]. By combing genetic screens with multi-omics analysis of matched PARP-sensitive and -resistance BRCA2-mutated mouse mammary tumors, PAR glycohydrolase (PARG) was found, the loss of which resulted in restoring PARylation formation and PARPi resistance [120]. Furthermore, the expression of PARP1 was significantly associated with PARPi toxicity. It has been revealed that both cells with low expression of PARP1 and cells harboring PARP1 LOF mutations were more resistant to PARPi [121, 122].

Pharmacological alteration also modulates PARPi inhibitor response. PARPi are substrates of multidrug resistance protein (MDR1, P-gp), encoded by ABCB1 gene [123]. Both in vivo and in vitro studies indicated the enhanced P-gp-mediated drug efflux contributed to the acquired resistance to PARPi [124, 125]. What’s more, the resistance could be reversed by coadministration of the P-gp inhibitors or genetic inactivation of P-gp [42, 123–125]. The overexpression of ABCB1 might be induced by long-term treating with PARPi but the mechanisms are still unclear. Compared to other factors, the weight of contribution in pharmacological changes to PARPi resistance in clinic is uncertain. More and more researches are needed to uncover the underlying mechanisms.

### Clinical implications towards PARPi resistance

To enhance PARPi sensitivity and overcome PARPi resistance, several feasible strategies should be considered and implemented in the future (Table 2): 1) PARPi-oncolytic herpes simplex viruses (oHSVs) combination; oHSVs, approved by FDA for recurrent melanoma, are genetically engineered to selectively kill cancer cells, due to their characteristics of amplifying and spreading within the tumor but not normal tissue. They are actively involved in manipulating DDR [126]. Recently, MG18L, a newly identified activity of oHSV, was reported to proteasomally degrade RAD51 and sensitize glioblastoma stem cells (GSCs) to PARPi killing in synthetic lethal-like fashion in vivo and in vitro. The combination of olaparib with MG18L greatly increased survival in both PARPi-sensitive and -resistant GSC-derived tumors. The combination therapy not only overcomes PARPi resistance but also expands its use to tumors with HR-proficient. Most importantly, oHSVs only infect and kill tumor cells but not normal cells compared to conventional medicines, which means that they may have fewer side effects [127]. Due to its broad anti-tumor efficacy in most solid tumors, this novel combination therapy should be applicable to other cancer stem cells and tumors; 2) PARPi-ionizing radiation (IR) combination; Nuclear localization is required for BRCA1 to participate in HR-mediated DNA repair [128]. IR can initiate the export of BRCA1 from the nucleus to the cytoplasm, leading to increased sensitivity of PARPi in wild-type BRCA1 and HR-proficient tumor cells [129, 130] However, because of the synthetic lethality of the combination therapy is p53-depend, it can only be used in wild-type p53 patients [131]. Meanwhile, PARPi induce radiosensitization in vitro and in vivo models [132]. What’s even more refreshing is HR restoration by 53BP1 pathway inactivation further increased radiosensitivity in preclinical model systems. It was showed that BRCA1-mutated tumors, which acquired drug resistance due to BRCA1-independent HR restoration, could be sensitized to radiotherapy [133]. In addition to the preclinical results, clinical studies were also attempted to exploit the efficacy of PARPi-IR combination. A phase 1, open-label dose escalation study (NCT00649207) evaluating veliparib in combination with whole brain radiation therapy (WBRT) in patients with brain metastases were originated with Mehta and his colleagues [134]. The preliminary efficacy results were better than predicted outcome based on the graded prognostic factors in the published nomogram. Based on encouraging safety and preliminary efficacy results, a randomized, controlled phase 2b study is ongoing. Other two phase 1 trials (NCT01264432, NCT01589419) indicated that the PARPi-IR combination treatment was well-tolerated and show good responses as well [135, 136]. Undoubtedly, further evaluation of PARPi-IR combination treatments is currently underway in multiple phase 2 clinical trials in patients with NSCLC and breast cancer (NCT02412371, NCT01386385, NCT01618357). 3) PARPi-CDKs inhibitors; DNA end resection is depended on cyclin-dependent kinases (CDKs) activity. A number of studies indicated that CDKs played important roles in PARPi resistance [36–41]. CDK inhibitor dinaciclib resensitized TBNC cells, which had acquired resistance to niraparib. In addition to TBNC cells, synthetic lethal strategy combining dinaciclib with niraparib was also highly efficacious in ovarian, prostate, pancreatic, colon, and lung cancer cells [137]. Currently, CDK12 attracted more attentions in PARPi resistance, due to its inactivating somatic alterations were recurrently observed in various cancers. Numerous evidences proved that CDK12 mutation or deficiency lead to cancer cells sensitivity to PARPi [37]. Furthermore, CDK12 inhibitors reversed de novo and acquired PARPi resistance in BRCA1-mutant breast cancer cells [39]. 4) PARPi-immunotherapy; Jiao et al and her colleagues revealed that PARPi upregulated PD-L1 expression in breast cancer cell lines via inactivating GSK3β, which in return leading to attenuate anticancer immunity. Moreover, the combination of PARPi and anti-PD-L1 therapy showed better therapeutic efficacy than each alone [138]. PARPi-mediated modulation of the immune response contributes to their therapeutic effects independently of BRCA1/2 mutations. Recently results suggested that PARPi promoted accumulation of cytosolic DNA fragments because of unresolved DNA lesions, which in turn activated the DNA-sensing cGAS-STING pathway and stimulated production of type I interferons to induce antitumor immunity independent of BRCAness [139]. At present, several clinical trials (NCT02734004, NCT03824704 and NCT02849496) are ongoing. In this term, all these trails may be informative. 5) PARPi-epigenetic drugs; As previously mentioned, epigenetic modification was associated with PARPi sensitivity [113, 117, 118]. Acetylation and deacetylation of histones is one of the most important mechanisms of posttranslational regulation of gene expression [140]. So far, numerous studies have declared that treating with histone deacetylation inhibitors (HDACi) and PARPi exhibited synergy effects due to the induction of HDACi on HRD, which as a result sensitized cancer cells to PARPi [141–144]. Several mechanisms have been observed. Firstly, it was reported that HDACi decreased the expression of DNA repair genes such as RAD51, CHK1, BRCA1 and RAD21 mediated through transcription factor E2F1 [145]. Secondly, HDACi blocked the deacetylation and expression of HSP90, resulting in the degradation of its substrates BRCA1, Rad52, ATR and CHK1 [146]. Finally, recent studies showed that acetylation blocked DNA damage-induced chromatin PARylation and HDACi treatment significantly increased the trapping of PARP1 at DSB sites in chromatin [147, 148]. Additionally, low doses of DNA methyltransferase inhibitor (DNMTi) induced BRCAness phenotype through downregulating expression of key HR genes [149]. The combination DNMTi and PARPi enhanced the cytotoxic effect by increasing the PARP “trapping” on DSB sites independent on BRCA mutations [150, 151]. However, there is no clinical trial to evaluate its effect until now. 6) PARPi-other drugs; In addition to the above mentioned, PARPi was also suggested to combinate with HSP90 inhibitors, ATR/CHK1 inhibitors and WEE1 inhibitors [152, 153]. BRCA1 function is reliant on HSP90. HSP90 inhibitor, 17-AAG, could induce HRD and increase Olaparib sensitivity of HR-proficient ovarian cancer cell lines [154]. Treating PARPi-resistant cells with 7-dimethylaminoethylamino-17-demethoxygeldanamycin, a HSP90 inhibitor, reversed the resistance state by decreasing the quantity of BRCA1 protein [92]. ATR/CHK1 and WEE1 have emerged as putative BRCAness factors that function in both checkpoint activation and in replication fork stability. ATR/CHK1 inhibitors and WEE1 inhibitors treatment were recently shown to reverse PARPi resistance in cancer cells [152]. Currently, several trails to the safety and efficacy of these combination treatments in sporadic cancers are in progress (NCT03579316, NCT04197713, NCT02576444, NCT02511795, NCT04065269, NCT03787680, NCT03330847, NCT03878095, NCT03462342, NCT03428607, NCT03682289). In a word, the combination therapy to overcome PARPi resistance and enhance PARPi sensitivity is still in its infancy and has a long way to go. More and more studies are needed to investigate the feasibility in clinic.

**Table 2:** The feasible combination therapy to enhance PARPi sensitivity and overcome PARPi resistance

Combination therapy	Trials	NCT	Phase	Treatment	Status	Study population
PARPi-oHSVs combination	No					
PARPi-IR combination	Yes	NCT00649207	I	Veliparib + WBRT^a^	Completed	Solid tumors with brain metastases
PARPi-IR combination	Yes	NCT01264432	I	Veliparib + IR	Completed	Peritoneal carcinomatosis; fallopian tube, ovarian and primary peritoneal cancers
PARPi-IR combination	Yes	NCT01589419	I	Veliparib + capecitabine + IR	Completed	Locally advanced rectal cancer
PARPi-IR combination	Yes	NCT02412371	I/II	Veliparib + Paclitaxel/Carboplatin + IR	Completed	Stage III NSCLC^b^
PARPi-IR combination	Yes	NCT01386385	I/II	Veliparib + Paclitaxel/Carboplatin + IR	Active, not recruiting	Stage III NSCLC
PARPi-IR combination	Yes	NCT01618357	I	Veliparib + IR	Recruiting	Breast cancer
PARPi-CDKi combination	No					
PARPi-immunotherapy	Yes	NCT02734004	I/II	Olaparib + MED14736	Active, not recruiting	Ovarian, breast, SCLC ^c^and gastric cancers
PARPi-immunotherapy	Yes	NCT03824704	II	Rucaparib + Nivolumab	Active, not recruiting	Epithelia ovarian cancer, fallopian tube cancer, primary peritoneal cancer, HGSC^d^ and endometrioid adenocarcinoma
PARPi-immunotherapy	Yes	NCT02849496	II	Olaparib + Atezolizumab	Recruiting	Locally advanced unresectable; metastatic non-HER2-positive breast cancer
PARPi- epigenetic drugs	No					
PARPi- HSP90 inhibitors	No					
PARPi-WEE1 inhibitors	Yes	NCT03579316	II	Olaparib + AZD1775	Recruiting	Recurrent fallopian tube, ovarian and primary peritoneal cancers
PARPi-WEE1 inhibitors	Yes	NCT04197713	I	Olaparib + AZD1775	Not yet recruiting	Advanced solid tumors with selected mutations and PARP Resistance
PARPi-WEE1 inhibitors	Yes	NCT02576444	II	Olaparib + AZD1775	Active, not recruiting	Tumors harboring either TP53 or KRAS mutations or mutations in KRAS and TP53
PARPi-WEE1 inhibitors	Yes	NCT02511795	I	Olaparib + AZD1775	Completed	Refractory solid tumors; Relapsed SCLC
PARPi-ATR inhibitors	Yes	NCT02576444	II	Olaparib + AZD6738	Active, not recruiting	Tumors harboring mutations leading to dysregulation of the PI3K/AKT pathway
PARPi-ATR inhibitors	Yes	NCT04065269	II	Olaparib + AZD6738	Recruiting	Gynaecological cancers
PARPi-ATR inhibitors	Yes	NCT03787680	II	Olaparib + AZD6738	Recruiting	Prostate cancer
PARPi-WEE1/ATR inhibitors	Yes	NCT03330847	II	Olaparib + AZD6738/ AZD1775	Recruiting	Metastatic triple negative breast cancer
PARPi-ATR inhibitors	Yes	NCT03878095	II	Olaparib + AZD6738	Recruiting	IDH1 and IDH2 mutant tumors
PARPi-ATR inhibitors	Yes	NCT03462342	II	Olaparib + AZD6738	Recruiting	HGSC
PARPi-ATR inhibitors	Yes	NCT03428607	II	Olaparib + AZD6738	Active, not recruiting	SCLC
PARPi-ATR inhibitors	Yes	NCT03682289	II	Olaparib + AZD6738	Recruiting	Clear cell renal cell cancer; Metastatic renal cell cancer; Metastatic urothelial cancer; Metastatic pancreatic cancer; Locally advanced pancreatic cancer

^a^WBRT: Whole Brain Radiation Therapy; ^b^NSCLC: Non-Small Cell Lung Cancer; ^c^SCLC: Small Cell Lung Cancer; ^d^HGSC: High Grade Serous Carcinoma

## Conclusions and perspectives

In the past few decades, PARPi was successfully developed in treating BRCA mutation patients, which provided proof-of concept that synthetic lethal interactions could be translated into cancer therapy. However, the preclinical and clinical investigation of PRARi is far from complete. In terms of PARPi resistance, multiple potential resistance mechanisms, such as HR restoration and protection of DNA replication fork have been identified. Nonetheless, the contribution weight of them to PARPi resistance is incomprehensible. Recently, the PRIMA trial results suggested that among patients with newly diagnosed advanced ovarian cancer who had a response to platinum-based chemotherapy, those who received niraparib had significantly longer progression-free survival (PFS) than those who received placebo, regardless of the presence or absence of HRD [155]. Based on it, we assumed that PARPi might kill cancer cells in ways other than DNA repair. The association between PARPi resistance and protection of DNA replication fork confirmed this conjecture. Therefore, we should comprehensively understand how PARPi functions, especially, how do the roles of PARPi in processes unrelated to DNA repair influence the anti-cancer activity of PARPi, which would be conductive to understand the development of resistance. Also, to overcome PARPi resistance and increase PARPi sensitivity, the optimal combination of PARPi and other treatment regimens are urgently needed to identify.

In addition to PARPi resistance, a serious of unanswered questions that could guide the optimal use of PARPi in the future, are not addressed. For example, what other proteins beyond BRCA1 and BRCA2 contribute to the efficacy of PARPi? Currently, PTEN has received a lot of attention as a promising biomarker to predicting the sensitivity of PARPi. PTEN is one of the tumor suppressor genes most frequently inactivated in human cancers [156]. It is reported that loss of PTEN lead to HRD, increased genomic instability and replication fork collapse [157–159]. At present, there is a growing body of preclinical evidence that tumors with loss of PTEN function are defective in HR and may, therefore, be hypersensitive to PARPi [159–161]. Likewise, there are lots of conflicting results that PTEN deficiency has no effect on PARPi sensitivity [162–164]. In a word, vulnerabilities of PTEN-deficient sporadic cancers to PARP inhibition remain controversial.

Besides, due to additional biological process beyond HR related to sensitivity of PARPi, we need to redefine the concert of concept of “BRCAness” and exploit new techniques of companion diagnostics to predict the response of patients to PARPi [24, 152]. Current BRCAnalysis assay could not effectively identify BRCAness. For example, genomic scars of BRCAness, as they are currently measured, probably reflect the alteration of the genome in the absence of HR over the entire lifetime of a tumor, they might not provide an accurate estimation of whether HR is still defective in tumor cells at the time that treatment is delivered. Other proposed approaches such as the use of mRNA expression signatures and the individual analysis of genetic alterations in HR-related genes are both lack of specificity. RAD51 accumulation and the formation of RAD51-ssDNA play key roles in both HR and protection of stalled DNA replication fork, therefore, RAD51 assay may be feasible in identifying PARPi-sensitive cancer patients and broadening the population who may be response to PRAPi-based therapy.

In conclusion, if all these issues can be figured out, we firmly believe that a substantial subset of cancer patients could benefit from PARPi.

## Data Availability

All the data obtained and/or analyzed during the current study were available from the corresponding authors on reasonable request.

## Abbreviations

PARP: Poly (ADP-ribose) polymerase
PARPi: PARP inhibitor
HR: Homologous recombination
HRD: Homologous recombination repair deficient
PARylation: ADP-ribosylation
DDR: DNA damage response
NHEJ: Non-homologous end joining repair
SSBR: Single stranded break repair
SSB: Single-strand breaks
BER: Base excision repair
gBRCAm: Germline mutations in BRCA1/2
sBRCAm: Somatic mutations of BRCA1/2
HGSOC: High-grade serious ovarian cancer
BRCAm: Mutation of BRCA1/2
MRN: Mre11-Rad50-Nbs1
ssDNA: Single-strand DNA
CDKs: Cyclin-dependent kinases
LOF: Loss-of functions
TNBC: Triple-negative breast cancer
LUAD: Lung adenocarcinoma
cfDNA: Circulating cell-free DNA
ORF: Open reading frame
BRCT: N-terminal domains of BRCA1
mCRPC: Metastatic castration-resistant prostate cancer
m^6^A: N^6^-methyladenosine
PARG: PAR glycohydrolase
MDR1: Multidrug resistance protein
oHSVs: Oncolytic herpes simplex viruses
GSCs: Glioblastoma stem cells
IR: Ionizing radiation
WBRT: Whole brain radiation therapy
HDACi: Histone deacetylation inhibitors
DNMTi: DNA methyltransferase inhibitor
PFS: Progression-free survival
